# The association between sport type and eating/body image concerns in high school students: a cross-sectional observational study

**DOI:** 10.1007/s40519-023-01570-3

**Published:** 2023-05-17

**Authors:** Giulio D’Anna, Lorenzo Lucherini Angeletti, Federica Benvenuti, Giulia Melani, Marco Ferroli, Francesca Poli, Rita Giulia Villano, Valdo Ricca, Francesco Rotella

**Affiliations:** Psychiatry Unit, Department of Health Sciences, University of Florence, Largo Brambilla, 3, 50134 Florence, Italy

**Keywords:** Adolescence, Eating disorders, Body image, Team sports, Inactivity

## Abstract

**Purpose:**

Disordered eating and body image concerns are increasingly common among adolescents, possibly representing the underpinning of eating disorders (EDs). This cross-sectional observational study aimed at investigating the relationship between various patterns of sports involvement or inactivity, and the abovementioned psychopathological dimensions.

**Methods:**

All adolescents attending their 3rd–5th Italian grade in a single high school reported their sociodemographic and anthropometric data, their weekly sports involvement, and filled the Eating Disorders Examination Questionnaire 6.0 (EDE-Q), the Body Uneasiness Test, and the Muscle Dysmorphia Disorder Inventory (for boys). Comparisons were performed considering sex, weekly hours of activity, and different sports type (none, individual, or team sports).

**Results:**

Of 744 enrolled students, 522 (70.2%) completed the survey. Girls showed higher underweight rates, preference for inactivity or individual sports, and higher psychometric scores compared to boys. Among girls, no differences were found based on time spent exercising or sports type. Inactive boys displayed worse weight- and shape-based psychopathology, higher body uneasiness, and higher appearance intolerance compared to those who devoted more time to exercise. Among boys, individual and team sports were associated with lower EDE-Q scores compared to inactivity, whereas body uneasiness and appearance intolerance were lower only in team sports.

**Conclusions:**

The study confirms the presence of remarkable sex differences in eating and body concerns of adolescents. Among boys, sports involvement is tied to lower ED psychopathology, and preference for team sports may be associated with reduced concerns. Wider longitudinal studies on will clarify the direction and specificity of these findings.

**Level of evidence:**

Level V—Cross-sectional observational study.

**Supplementary Information:**

The online version contains supplementary material available at 10.1007/s40519-023-01570-3.

## Introduction

Eating disorders (EDs) constitute a significant epidemiological and clinical challenge, and their onset during adolescence has become increasingly common [1]. Despite the well-acknowledged major prevalence of these conditions among young women, there is growing awareness about the occurrence of ED psychopathology among male adolescents [2, 3]. With respect to the Italian population, recent epidemiological studies found that a high proportion of young people of both sexes may be at risk of EDs [4], and that the percentage of adolescents reporting an under- or over-weight condition is increasing [5].

Inactivity and sedentary behaviour have been reported to be strongly associated with adverse physical and mental health outcomes, including the development of ED symptoms [6, 7]. For this reason, moderate-to-high physical activity is encouraged to promote general and mental health [8]; [9]. On the other side, large amounts of time devoted to compulsive exercise are known to constitute a nuclear aspect of overt ED psychopathology [10]. These observations suggest the need for large-scale evaluations to effectively screen adolescents at high risk to develop an ED with respect to different patterns of physical activity. In this framework, the evaluation of the types of sport practiced—rather than merely the time devoted to it—may provide relevant information [11]. In fact, both in adult and adolescent populations, various sport activities proved to be differentially associated with ED psychopathology [12–14], and they have been suggested to act as both risk or protective factors for pathological eating and body image concerns [15, 16]. More in detail, the specific physical requirements of some disciplines could be associated with higher distress levels regarding diet, body shape and weight [17], and individual or aesthetic activities promoting muscularity or leanness have been associated with pathological eating and body attitudes [13, 18]. A sex-specific and potentially under-addressed facet in the continuum of disordered eating and body-centred behaviour is represented by muscle dysmorphia in male adolescents (MD, a pathological preoccupation with one self’s muscularity and leanness) [3, 19, 20]. It must be highlighted that further facets of ED psychopathology may be tied to gender identity or sexual orientation [21] rather than biological sex [22], but the present study will focus on the latter, which is mostly addressed by research on this topic, and the terms “boys” and “girls” should be interpreted accordingly throughout the text.

Most of the available studies addressing the relationship between physical activity and ED psychopathology are based on the “leanness” vs “non-leanness” sports paradigm [23]. On the contrary, the “team” vs “individual” sports perspective is under-addressed in representative, non-clinical populations, and it has led to inconclusive or mixed results which warrant further evaluation [24–26]. In fact, the preference for sedentary behaviours or individual training may indirectly reflect a rise in extrinsic motivations to practice sports, a condition that has been identified as a risk factor for EDs [27], and the internalisation of appearance ideals may be tied to disordered eating, especially among boys [28]. Conversely, engagement in team sports may be associated with lower disordered eating symptoms [25], possibly due to its cooperative goals—e.g., in ball games—as opposed to activities meant to optimise individual performances or appearance.

Overall, data on the topic remain controversial, ranging from higher to lower psychopathology levels among athletes as compared to non-athletes, and only a few studies tried to investigate the possible role of specific sport types in this complex scenario [13, 14]. The present work aimed to investigate ED symptomatology and body image discomfort in a representative sample of high school students, to explore:

- the relationship between weekly time spent doing any sport and eating/body image concerns;
- the relationship between sport type (none, individual, or team sport) and eating/body image concerns.

## Materials and methods

The present observational, cross-sectional study involved all the students attending the 3rd–5th grade (age range 16 to 19) of a public high school in Florence (Italy), and it was carried out in November–December 2021. The investigators decided a priori to target the general population, meaning that no preliminary screening for EDs was carried. Before enrolment, all potential participants and their parents or legal guardians received an informative paper about the survey, and a further explanatory video which was made available on the website of the high school. Subsequently, participants (and their parents or legal guardians, for individuals under 18) provided written informed consent to participate. For each class, the survey was filled out by all the enrolled subjects on a single occasion, during school hours. The study protocol was approved by the Committee for the Ethics of Research of the University of Florence (protocol number 0027558, 13/02/2020), and the confidentiality of students was always ensured. The study was performed in accordance with the ethical standards as laid down in the 1964 Declaration of Helsinki and its later amendments.

### Assessed variables

Participants provided their age, sex, and their current height and weight. Body Mass Index (BMI, in kg/m^2^) was obtained from self-reported height and weight, and categorised into three groups (underweight, normal weight, and overweight), using age- and sex-based normative centiles [29, 30] (see also Supplementary Table 2 for the age range of the study sample). Subjects were also asked: “Do you practice sport on a regular basis (i.e., at least once a week)?”, what was the main activity practiced, whether it was undertaken at an individual or team level, and the weekly number of hours spent in sport activities. The latter were categorised into four groups: no sport, and low, intermediate, and high activity groups, based on sex-specific tertile values of individuals who practiced any sport, similar to previous studies addressing physical activity among adolescents and young adults, and treating the inactive subgroup as a separate category [31, 32].

The following psychometric tests were administered.

- Eating Disorders Examination Questionnaire 6.0 (EDE-Q 6.0) [33], a 28-item test to assess the core psychopathology of EDs, which provides four subscales (dietary restraint, eating concern, weight concern, shape concern) and a total score obtained by averaging their scores with excellent reliability (Cronbach's *α* = 0.97) in the Italian adaptation [33].
- Body Uneasiness Test (BUT) [34], a 71-item self-report that consists of two parts: BUT-A, evaluating discomfort with one self’s body, and BUT-B, examining worries about specific body parts or functions. The 34-item BUT-A scale was used in the present study: it provides five subscales (weight phobia, body image concern, avoidance, compulsive self‐monitoring, depersonalisation), and a total score (global severity index, GSI) obtained by averaging the scores of all items. In the Italian validated adaptation, reliability coefficients of the scales ranged from 0.79 and 0.90, showing excellent internal consistency, good test–retest reliability (as the correlation coefficients were greater than 0.7), as well as a good predictive validity for anorexia nervosa (restrictive and binge-purging types), and for bulimia nervosa [34]. BUT-B items and subscales refer to specific body parts and functions which can not be directly linked to physical activity, whereas BUT-A directly evaluates concerns regarding body image dissatisfaction: therefore, BUT-A scores were the outcome of interest for the present study.
- Muscle Dysmorphia Disorder Inventory [35] (in the male subgroup), which includes 13 questions assessing three dimensions: drive for size, appearance intolerance, perceived functional impairment; the total score is obtained as the sum of the abovementioned items. The Italian validated adaptation showed an excellent reliability (Cronbach's *α* = 0.85) [35].

Single participant data were analysed if 85% of responses had been provided; single-item missing data were calculated as average of the items concurring to the corresponding subscale.

### Statistical analyses

Categorical variables were reported as absolute and relative frequency. Continuous variables were reported as mean ± standard deviation. Tertile cutoff values for weekly time of physical activity were provided to categorise this specific variable for each sex. For between-group comparisons, chi-square test (*χ*^2^), Student’s *t* test, or age- and BMI-adjusted one-way ANCOVA (analysis of covariance) with Bonferroni post-hoc test for multiple comparisons were used. Effect sizes for the abovementioned tests were also evaluated (Cramer’s *V*, Cohen’s *d*; and *η*^*2*^, respectively). Analyses were performed with the Statistical Package for Social Sciences 25.0 [36]. The null hypothesis was rejected for *α* < 0.05.

## Results

Among 744 potential participants, 342 (46.0%) were girls, and 402 (54.0%) were boys. A total of 522 individuals (70.2%) provided written informed consent and completed the survey. The responding sample consisted of 232 girls (44.4%) and 290 boys (55.6%). The non-responding sample (*n* = 222) consisted of 110 girls (49.5%) and 112 boys (50.5%). Responding and non-responding subjects were balanced with respect to the publicly available information on classes composition: age (16.90 ± 0.80 and 16.98 ± 0.82, respectively; *t* = 1.24, *p* = 0.431), and sex (*χ*^2^ = 1.63, *p* = 0.201).

Of the 522 participants, 507 subjects reported both their height and weight, and were, therefore, included in analyses involving BMI. Of them, 57 (11.2%) were underweight, 412 (81.2%) had a normal weight, and 38 (7.5%) were overweight or obese. The average BMI was 20.89 ± 2.54 kg/m^2^. Individuals who practiced sports were 417 (79.9%), and they reported to exercise for an average time of 5.69 ± 3.11 h per week. Among active subjects, 278 (66.7%) practiced an individual sport, whereas 139 (33.3%) practiced a team sport (see Supplementary Table 1 for details).

The characteristics of the subgroups of girls and boys are summarised in Table 1. Overall, girls had a lower BMI and higher rates of underweight, as defined by age- and sex-specific normative centiles. Boys reported to practice physical activity for a higher amount (6.18 ± 3.21 h, tertiles cutoffs: 5 h and 7 h) compared to girls (4.94 ± 2.79 h, tertiles cutoffs: 3.5 h and 6.5 h, respectively) (*t* = − 4.07, *p* < 0.001), and a significantly higher proportion of girls did not practice any sport or did it at an individual level. Among girls, higher psychopathology scores were observed in all the scales administered.

**Table 1:** Characteristics of subsamples based on sex, with age- and BMI-adjusted comparisons

	Girls (*n* = 232)		Boys (*n* = 290)		*t*(522)^a^	*p*	*d*
	*M*	SD	*M*	SD			
Age	16.97	0.82	16.99	0.81	− 0.22	.820	− 0.02
BMI	20.25	1.99	21.37	2.80	− 5.05	< .001	− 0.45
EDE dietary restraint	1.68	1.62	0.54	0.85	10.29	< .001	0.91
EDE eating concern	1.41	1.34	0.42	0.69	10.82	< .001	0.95
EDE weight concern	2.29	1.73	0.78	0.95	12.63	< .001	1.11
EDE shape concern	2.75	1.74	1.01	1.11	13.79	< .001	1.21
EDE total score	2.03	1.48	0.69	0.75	13.38	< .001	1.18
BUT weight phobia	2.21	1.32	0.90	0.80	13.93	< .001	1.23
BUT body image concern	1.88	1.28	0.84	0.87	10.86	< .001	0.95
BUT avoidance	0.74	0.92	0.16	0.32	9.90	< .001	0.87
BUT compulsive self-monitoring	1.69	1.15	0.79	0.72	10.89	< .001	0.96
BUT depersonalisation	1.20	2.83	0.36	1.52	4.31	< .001	0.38
BUT global severity index	1.57	1.07	0.63	0.56	12.99	< .001	1.14
MDDI drive for size	–	–	10.98	4.74	–	–	–
MDDI appearance intolerance	–	–	6.09	2.95	–	–	–
MDDI functional impairment	–	–	6.52	3.56	–	–	–
MDDI total score	–	–	23.60	8.03	–	–	–

		Girls (*n* = 232)		Boys (*n* = 290)		**χ**^**2**^	*p*	*V*
		*n*	*%*	*n*	*%*			
Weight	Underweight	34	15.3%	23	8.0%	17.95	< .001	0.19
	Normal weight	182	81.9%	230	80.8%			
	Overweight	6	2.7%	32	11.2%			
Weekly hours of activity	No sport	65	28.0%	40	13.8%	49.40	< .001	0.31
	Lower tertile	83	35.8%	56	19.3%			
	Intermediate tertile	39	16.8%	100	34.5%			
	Upper tertile	45	19.4%	94	32.4%			
Sport type	No sport	65	28.0%	40	13.8%	40.70	< .001	0.28
	Individual sport	135	58.2%	143	49.3%			
	Team sport	32	13.8%	107	36.9%			

*M* mean, *SD* standard deviation, *d* Cohen’s *d*, *V* Cramer’s *V*, *BMI* body mass index, *EDE* Eating Disorder Examination Questionnaire, *BUT* body uneasiness test, *MDDI* muscle dysmorphia disorder inventory

^a^*N* = 507 (285 boys, 222 girls) for analyses involving BMI

Given the significant differences between boys and girls for almost all the variables investigated, we split our sample for sex. Each group was then divided into four subgroups based on sex-specific tertiles of the hours of activity per week as follows: “No sport”, “Lower tertile”; “Intermediate tertile”, “Higher tertile”.

Comparisons among subgroups are presented in Tables 2 and 3 for girls and boys, respectively. Among boys, BMI showed a different distribution among groups: more in detail, inactive subjects displayed a lower BMI compared to the upper tertile. No differences were observed among subgroups for girls. In males, EDE-Q weight concern, shape concern, and total scores had a different distribution among groups: boys not doing sports had higher shape concern and total scores when compared to the two upper tertiles, and higher weight concern scores compared to the intermediate tertile. Regarding BUT scores, only body image concern had a different distribution among subgroups, and it proved to be worse in the inactive group of boys as compared to the upper tertile. Appearance intolerance scores were lower in the intermediate tertile when compared both to the inactive group and the lower tertile, whereas the upper tertile show lower scores compared to the inactive group. Finally, a higher perceived functional impairment was observed in the upper tertile compared to the other subgroups.

**Table 2:** Girls: characteristics and age- and BMI-adjusted comparisons of subgroups based on time of activity

*Girls*	No sport (*n* = 65)		Lower tertile (*n* = 83)		Intermediate tertile (*n* = 39)		Upper tertile (*n* = 45)		*F*(1,232)^a^	*η*^*2*^
	*M*	SD	*M*	SD	*M*	SD	*M*	SD		
Age	17.00	0.10	16.92	0.10	17.07	0.11	16.86	0.12	1,00	.01
BMI	20.06	0.25	20.33	0.25	20.22	0.27	20.46	0.31	0.68	.01
EDE dietary restraint	1.67	0.20	1.58	0.19	1.96	0.21	1.30	0.24	1.23	.01
EDE eating concern	1.33	0.17	1.37	0.16	1.56	0.18	1.33	0.20	0.27	.00
EDE weight concern	2.38	0.21	2.20	0.20	2.38	0.22	2.14	0.25	0.59	.01
EDE shape concern	3.02	0.21	2.54	0.21	2.95	0.22	2.31	0.25	1.34	.02
EDE total score	2.10	0.18	1.92	0.18	2.21	0.19	1.77	0.22	0.72	.01
BUT weight phobia	2.33	0.16	2.04	0.16	2.49	0.17	1.94	0.20	0.57	.01
BUT body image concern	2.14	0.15	1.78	0.15	1.97	0.16	1.50	0.18	1.73	.02
BUT avoidance	0.85	0.11	0.75	0.11	0.77	0.12	0.50	0.14	1.05	.01
BUT compulsive self-monitoring	1.80	0.14	1.54	0.14	1.78	0.15	1.58	0.17	0.76	.01
BUT depersonalisation	1.05	0.37	1.63	0.36	1.24	0.39	0.70	0.44	1.22	.01
BUT global severity index	1.71	1.06	1.63	1.07	1.53	1.11	1.33	1.01	1.21	.01

*M* mean, *SD* standard deviation, *F* *F* test, *η*^*2*^ effect size for the ANOVA model, *BMI* body mass index, *EDE* Eating Disorders Examination Questionnaire, *BUT* body uneasiness test

^a^*N* = 222 for analyses involving BMI

**Table 3:** Boys: characteristics and age- and BMI- adjusted comparisons of subgroups based on time of activity

*Boys*	No sport (*n* = 40)		Lower tertile (*n* = 56)		Intermediate tertile (*n* = 100)		Upper tertile (*n* = 94)		*F*(1,290)^a^	*η*^*2*^
	*M*	SD	*M*	SD	*M*	SD	*M*	SD		
Age	17.25	0.12	16.95	0.90	17.03	0.09	16.85	0.08	2.83*	.03
BMI	20.21	0.45	21.01	0.31	21.90^†^	0.31	21.71^†^	0.29	3.59**	.04
EDE dietary restraint	0.71	0.13	0.47	0.09	0.52	0.09	0.55	0.08	0.52	.00
EDE eating concern	0.62	0.11	0.44	0.07	0.35	0.07	0.39	0.07	0.21	.00
EDE weight concern	1.20	0.15	0.82	0.10	0.61^‡^	0.10	0.74	0.09	1.43*	.01
EDE shape concern	1.58	0.17	1.14	0.11	0.79^‡^	0.12	0.89^‡^	0.11	1.80**	.02
EDE total score	1.03	0.11	0.72	0.08	0.57^‡^	0.08	0.64^†^	0.07	1.04**	.01
BUT weight phobia	1.08	0.13	0.95	0.08	0.86	0.08	0.86	0.08	0.20	.00
BUT body image concern	1.18	0.14	0.96	0.09	0.75	0.09	0.72^†^	0.90	1.19*	.01
BUT avoidance	0.23	0.05	0.20	0.03	0.13	0.03	0.13	0.03	0.65	.01
BUT compulsive self-monitoring	0.83	0.12	0.77	0.08	0.75	0.08	0.85	0.07	0.34	.00
BUT depersonalisation	0.43	0.26	0.27	0.17	0.52	0.17	0.29	0.16	0.25	.00
BUT global severity index	0.70	0.67	0.68	0.62	0.58	0.50	0.62	0.55	0.57	.01
MDDI drive for size	11.60	5.77	11.19	4.99	10.73	4.30	10.87	4.60	0.37	.00
MDDI appearance intolerance	6.92	3.67	6.76	3.45	5.65^‡§^	2.24	5.80^†^	2.86	3.14***	.03
MDDI functional impairment	4.95	2.02	6.10	2.77	5.91	2.83	8.08 ^‡‡§§$^	4.53	10.89***	.10
MDDI total score	23.47	9.38	24.07	7.92	22.29	6.73	24.76	8.64	1.62	.02

*M* mean, *SD* standard deviation, *F* *F* test, *η*^*2*^ effect size for the ANOVA model, *BMI* body mass index, *EDE* Eating Disorders Examination Questionnaire, *BUT* body uneasiness test, *MDDI* muscle dysmorphia disorder inventory

^a^*N* = 285 for analyses involving BMI

^*^: *p* < 0.05; **: *p* < 0.01; ***: *p* < 0.001

Post-hoc difference from the "No sport" group: ^†^, *p* < 0.05; ^‡^, *p* < 0.01; ^‡‡^, *p* < 0.001

Post-hoc difference from the "Lower tertile" group: ^§^, *p* < 0.05, ^§§^, *p* < 0.01

Post-hoc difference from the "Intermediate tertile" group: ^$^, *p* < 0.001

The assessment of subgroups based on the type of sport practiced is shown in Tables 4 and 5 for girls and boys, respectively. In girls, no comparison reached statistical significance, although systematically lower scores could be observed in team sports. Among boys, different distributions were observed for age, BMI, and part of the psychometric indexes: EDE-Q (weight concern, shape concern, and total score), BUT (body image concern and GSI), MDDI (appearance intolerance and functional impairment). More in detail, subjects who did not practice any sport had a higher age when compared to participants in team sports, and they had lower BMI, higher EDE-Q weight concern, shape concern, and total scores compared to subjects of both sports categories. Moreover, the team sport subgroup displayed lower BUT body image concern and GSI scores, and a lower MDDI appearance intolerance, when compared to the “No sport” subgroup. MDDI functional impairment was higher in individual sports than in the inactive group. Even if no differences were detected between team and individual sports for any of the variables investigated, higher significance levels and effect sizes were observed for the team sports vs inactivity comparisons, as compared to the corresponding analysis for individual sports.

**Table 4:** Girls: characteristics, and age- and BMI-adjusted comparisons of subgroups divided by sport type

*Girls*	No sport (*n* = 65)		Individual sport (*n* = 135)		Team sport (*n* = 32)		*F*(1,290)^a^	*η*^*2*^
	*M*	SD	*M*	SD	*M*	SD		
Age	17.00	0.84	16.96	0.82	16.97	0.82	0.06	.00
BMI	20.06	2.04	20.29	1.96	20.48	2.03	0.50	.00
EDE dietary restraint	1.64	1.69	1.76	1.64	1.15	1.11	1.87	.02
EDE eating concern	1.30	1.26	1.51	1.38	1.09	1.31	1.59	.01
EDE weight concern	2.33	1.77	2.33	1.69	1.95	1.69	0.87	.01
EDE shape concern	2.98	1.76	2.71	1.74	2.32	1.64	1.77	.01
EDE total score	2.06	1.50	2.08	1.49	1.63	1.33	1.52	.01
BUT weight phobia	2.31	1.36	2.25	1.32	1.82	1.27	1.42	.01
BUT body image concern	2.10	1.27	1.83	1.31	1.56	1.16	1.79	.01
BUT avoidance	0.83	0.90	0.74	0.98	0.50	0.63	0.68	.01
BUT compulsive self-monitoring	1.79	1.13	1.71	1.17	1.28	1.03	2.60	.02
BUT depersonalisation	1.04	1.02	1.38	3.64	0.69	0.81	0.73	.00
BUT global severity index	1.70	1.05	1.58	1.10	1.24	0.91	1.75	.01

*M* mean, *SD* standard deviation, *F* *F* test, *η*^*2*^ effect size for the ANOVA model, *BMI* body mass index, *EDE* eating disorders examination questionnaire, *BUT* body uneasiness

^a^*N* = 222 for analyses involving BMI

**Table 5:** Boys: characteristics, and age- and BMI-adjusted comparisons of subgroups divided by sport type

*Boys*	No sport (*n* = 40)		Individual sport (*n* = 143)		Team sport (*n* = 107)		*F*(1,232)°	*η*^*2*^
	*M*	*SD*	*M*	*SD*	*M*	*SD*		
Age	17.25	0.90	17.00	0.77	16.87^†^	0.80	3.30*	.02
BMI	20.21	3.12	21.52^†^	2.58	21.59^†^	2.90	3.77*	.03
EDE Dietary Restraint	0.56	0.92	0.52	0.74	0.56	0.96	0.06	.00
EDE Eating Concern	0.50	0.84	0.40	0.74	0.43	0.58	0.29	.00
EDE Weight Concern	1.05	1.17	0.73^†^	0.95	0.76^†^	0.88	1.21*	.01
EDE Shape Concern	1.36	1.42	1.04^†^	1.09	0.87^‡‡^	1.01	2.37***	.02
EDE Total Score	0.87	0.96	0.67^†^	0.74	0.66^‡^	0.70	0.83*	.01
BUT Weight Phobia	0.95	0.93	0.95	0.82	0.84	0.71	0.49	.00
BUT Body Image Concern	1.05	1.06	0.85	0.82	0.78^†^	0.88	1.06*	.01
BUT Avoidance	0.20	0.40	0.17	0.35	0.14	0.26	0.34	.00
BUT Compulsive Self-Monitoring	0.81	0.80	0.86	0.75	0.71	0.66	1.21	.01
BUT Depersonalisation	0.40	0.68	0.29	0.48	0.46	2.42	0.41	.00
BUT Global Severity Index	0.72	0.68	0.66	0.58	0.57^†^	0.50	0.93*	.01
MDDI Drive for Size	11.78	5.70	11.08	4.66	10.68	4.45	0.56	.00
MDDI Appearance Intolerance	6.86	3.51	6.14	2.86	5.71^‡^	2.75	2.48**	.02
MDDI Functional Impairment	5.02	2.08	6.85^†^	3.64	6.72	3.77	4.65*	.03
MDDI Total Score	23.67	9.17	24.08	7.91	23.12	7.71	0.36	.00

*M*, mean; *SD*, standard deviation; *F*, *F* test; *η*^*2*^, effect size for the ANOVA model; BMI, body mass index; EDE, Eating Disorders Examination Questionnaire; BUT, Body Uneasiness Test; MDDI, Muscle Dysmorphia Disorder Inventory

^*^: *p* < 0.05; **: *p* < 0.01; ***: *p* < 0.001

°: N = 285 for analyses involving BMI

*Post-hoc* difference from the “No sport” group: ^†^, *p* < 0.05; ^‡^, *p* < 0.01; ^‡‡^, *p* < 0.001

## Discussion

The present study, conducted on a representative sample of high school students, shows a strong asymmetry between girls and boys for the dimensions investigated. In fact, girls reported a remarkably lower BMI (15.3% of them were underweight), and higher ED symptomatology and body uneasiness. Girls were also less likely to be involved in sport activity or practiced it more commonly at an individual level and for a lower amount of time. These observations, tied with data derived from large-scale epidemiological studies in the same geographic area [4, 5], underline the need for thorough screening, prevention programs, and targeted early intervention for EDs among female adolescents [6, 37, 38].

It is well known that patients with full-blown EDs often spend significant amounts of time performing physical activity as a maintaining factor of their symptomatology [39]. The present findings showed that, in a non-clinical adolescent sample, higher amounts of time practicing sports were not associated with worse psychometric scores among girls, and it was rather associated with lower scores among boys. This observation suggests that the promotion of physical activity among adolescents should be at least non-harmful. However, it should be noted that athletes usually display a higher tendency towards compulsive exercising compared to non-athletes [10] and that the relationship between physical activity and ED symptoms could be bidirectional (i.e., boys with higher levels of body image and eating concerns could be less prone to practice physical activity). For these reasons, the topic needs further investigation, and longitudinal studies on wider samples are needed to better understand all the possible clinical implications of these results.

In a screening-oriented approach, the present study indirectly suggests the opportunity to include lack of physical activity and sedentariness among the dimension investigated for the early detection of poor general and mental health among adolescents [6]. Previous studies outlined that body image discomfort, appearance dissatisfaction, and psychological distress often precede the onset of EDs, and that these dimensions are over-represented among sedentary individuals [40, 41]. Notably, it has been also reported that disordered eating behaviour leads to poor self-rated health, greater amount of leisure sitting time, and non-health-related motives to exercise [7]. Regarding the latter point, a large European Union-based study reported that women tend to choose sport to ameliorate their self-esteem, whereas men more frequently report a purpose of social integration [42]. It could, therefore, be hypothesised that the relationship between disordered eating and physical activity displays differential trajectories in males and females.

The comparison between lack of involvement in any sport, practicing an individual sport, and practicing a team one, showed a clear pattern of distribution in sex-based subgroups. This topic is poorly explored in literature and allowed us to integrate different facets of the role of physical activity on mental health [24–26]. In the study sample, a significant overlap existed between team sports and non-leanness sports (with a higher importance of technical skills), and between individual sports and leanness sports. Therefore, future studies must selectively investigate and consider this possible confounding factor. Nevertheless, the present data outline clear patterns of results which warrant attention per se. In fact, ED symptoms and body uneasiness indexes did not remarkably differ among the three subgroups in girls, suggesting a lower impact of the type of physical activity, as seen for exercise time. Conversely, among boys, subjects with sports involvement displayed significantly lower distress regarding shape, weight, and body image, when compared to subjects with a lack of sports involvement. The only major exception to this pattern regarded the functional impairment subscale of MDDI, with higher scores among those who practiced individual sports or engaged in physical activity for a higher amount of time. This finding may be explained by the fact that subjects who devote more hours to sports activity and/or perform it individually have less time for other study or leisure activities and could perceive less social involvement. MD-related body image dissatisfaction is known to constitute a potential concern in non-clinical samples of male bodybuilders and gym users [43, 44], and the observed trend of perceived functional impairment possibly identifies an individual, performance-related feature, which may exert a negative impact of other aspects of one’s personal life.

With different psychological and socio-cultural factors predisposing to disordered eating [16, 45], the accent on physical appearance and the related lifestyle may constitute an extrinsic motivation to do physical activity—a factor which already proved to be associated with higher EDs risk [7, 46]. Our male sample showed that team sports were associated with lower body uneasiness when compared to inactivity. A possible explanation lies on the fact that team sports are usually based on achievements that transcend the above-mentioned individual goals. Even if team environment may exert both a protective or detrimental role on psychological well-being and on ED symptoms of young athletes [47, 48], cooperative sports are reported to be generally associated with lower anxiety and depression levels as compared to individual activities [49]. The team sports mostly practiced in our sample were ball games, which are only marginally associated with body and eating-centred behaviour compared to other forms of activities, i.e., aesthetic, weight-dependent, endurance, power, and technical sports, which are more frequently undertaken at an individual level [23, 50]. In fact, individual sports emphasising thinness and muscularity have already been proposed to be associated with pathological eating and body attitudes [18, 51]. Besides, the increasing popularity of weight-sensitive sports with “body paradigms” should be evaluated accordingly, as they may exert a detrimental action on the otherwise desirable role of sports on physical and mental health [8, 9, 52]. In this light, regardless of sex-specific risk factors, the reduction of weight- and appearance-related activities could exert some protection against the onset of EDs [53].

Overall, our data seem to highlight a clear, different distribution of psychopathological distress between inactive and active subgroups for almost all the variables investigated. Among girls, these observations did not reach statistical significance. On one hand, it could be hypothesised that physical activity in women has a weaker differential impact on ED psychopathology, given the major role of other risk factors. On the other hand, it should be noted that the number of women that performed a team sport was significantly lower, compared to boys (32 vs 107), and this could have affected the analyses presented. Among boys, sport activity may display a protective role with respect to ED symptomatology, without detectable limits concerning the number of hours devoted to these activities. The design of the present study does not allow to make inferences on the possible negative role of individual activities. In addition, the current study design did not combine the type of sport and the time devoted to it, meaning that—in different subgroups based on sports type—higher amounts of time spent in these activities may play a differential role. It is possible to hypothesise that psychological and interpersonal factors may influence distress patterns through sports preferences. Even if no differences were detected between individual team sports, the lower scores and greater effect sizes observed in adolescents involved in team sports—compared to inactivity—suggest that cooperative team sports may identify individuals who express less body and diet-centred concerns, and who are, therefore, at lower risk for EDs symptomatology.

### Strength and limits

Given these preliminary findings, the conceptualisation provided in the present study (i.e., the accent on the individual or team dimension) may constitute an alternative and valuable point of view as compared to the well-established “leanness” vs “non-leanness” paradigm [50, 51], warranting further exploration in future studies for its potentially significant epidemiological and clinical implications. It is noteworthy that most of the team sports practiced were non-leanness activities, mostly based on technical skills, whereas individual sports substantially overlapped with leanness sports. Hence, a major limitation of the present study is the possible confounding role of these specific sports characteristics. Future studies focusing on non-leanness individual sports and leanness team sports will be able to disentangle this relationship, and to confirm the innovativeness of the proposed paradigm.

Further limitations of the present study must be acknowledged. First, the self-report nature of the investigation may expose data to minor inaccuracies—e.g., the fact that adolescents tend to overestimate their height and underestimate their weight [54], that athletes may undervalue their disordered eating when assessed through a self-report investigation [15], and that the weekly time spent in these activities is only an indirect estimate of sports intensity. Second, the sample size was not adequate to perform multivariate analyses, and it could be speculated that smoother differences for psychometric scales among females were not detected due to low statistical power. In addition, the small sample size made did not allow to combine the analysis of sports type and weekly exercise time, nor to conduct separate analyses for smaller subgroups (e.g., a putative minority of subjects practicing massive physical activity, rather than simply performing the tertile-based assessments). The non-response rate (29.8%) is in line with other large-scale, self-reported surveys conducted on the general population [55], and no differences were found in age and sex between responding and non-responding subjects. For this reason, to achieve higher statistical power, it seems necessary to expand the catchment area (e.g., with a multi-centre approach). Another limitation of the present study lies on limit that all subjects enrolled attend the same science high school, and in a narrow age range. Therefore, our data cannot be generalised to other socio-cultural contexts and to a broader age group. Moreover, it was not possible to retrieve other socio-demographic information, such as gender, sexual orientation, ethnicity, and socio-economic status, which may have acted as confounding factors (e.g., different accessibility to sports facilities, or high school choice) [56]. The cross-sectional design of the present study does not allow to draw inferences on the causal direction of the associations observed. Longitudinal studies, with a larger sample size including a larger age range, and with participants drawn from different catchment areas are needed to better describe all the different patterns of association between specific sport activities and ED symptomatology.

### What is already known on this subject?

Disordered eating and body image concerns among adolescents are common and exhibit a sex-specific distribution. Sports involvement and inactivity have been variously associated with these conditions and with differential eating disorders (ED) risk—mostly addressed through the “leanness” vs “non-leanness” sport paradigm.

### What this study adds?

The conceptualisation provided in the present study (i.e., the accent on the individual or team sports dimension) may constitute an alternative and valuable point of view to promote physical and mental health of adolescents. Even if ED-related concerns are mostly tied to inactivity, team sports involvement may exert an additional protective value, whose epidemiological and clinical implications need to be evaluated in longitudinal studies.

## Supplementary Information

Below is the link to the electronic supplementary material.

Supplementary file1 (DOCX 16 KB)

## Data Availability

The data that support the findings of this study are available from the corresponding author, upon reasonable request.
