# Supplementary material for: The association between sport type and eating/body image concerns in high school students: a cross-sectional observational study
Source: Eat Weight Disord. 2023 May 17;28(1):43. doi: 10.1007/s40519-023-01570-3 (PMC10191999; doi:10.1007/s40519-023-01570-3)
Supplement: Supplementary file 1 — Supplementary file1 (DOCX 16 KB) [file 40519_2023_1570_MOESM1_ESM.docx]

ADDITIONAL MATERIAL

| **N = 522** | **n (%)** |
| --- | --- |
| **None** | 105 (20.1%) |
| **Individual sports** | |
| Gym | 59 (11.3%) |
| Tennis | 53 (10.2%) |
| Martial arts | 34 (6.5%) |
| Athletics | 26 (5.0%) |
| Dance | 25 (4.8%) |
| Swimming | 24 (4.6%) |
| Artistic gymnastics | 18 (3.4%) |
| Cycling | 11 (2.1%) |
| Free body gymnastics | 10 (1.9%) |
| Other | 18 (6.5%) |
| **Team sports** | |
| Football | 68 (13.0%) |
| Volleyball | 34 (6.5%) |
| Basketball | 24 (4.6%) |
| Water polo | 6 (1.1%) |
| Other | 7 (5.0%) |

**Table S1**. Detail of sports practiced in the respondent sample.

| **Age** | **Boys** | | **Girls** | |
| --- | --- | --- | --- | --- |
|  | **Underweight** | **Overweight** | **Underweight** | **Overweight** |
| **16.0** | 17.54 | 23.90 | 17.91 | 24.37 |
| **16.5** | 17.80 | 24.19 | 18.09 | 24.54 |
| **17.0** | 18.05 | 24.46 | 18.25 | 24.70 |
| **17.5** | 18.28 | 24.73 | 18.38 | 24.85 |
| **≥ 18** | 18.50 | 25.00 | 18.50 | 25.00 |

**Table S2**. Sex-specific, age-based body mass index cut-offs used in the study sample.
